# The effectiveness and cost-effectiveness of opportunistic screening and stepped care interventions for older hazardous alcohol users in primary care (AESOPS) – A randomised control trial protocol

**DOI:** 10.1186/1472-6963-8-129

**Published:** 2008-06-12

**Authors:** Simon Coulton, Jude Watson, Martin Bland, Colin Drummond, Eileen Kaner, Christine Godfrey, Alan Hassey, Veronica Morton, Steve Parrott, Tom Phillips, Duncan Raistrick, Daphne Rumball, Gillian Tober

**Affiliations:** 1 Centre for Health Service Studies, University of Kent, Canterbury, UK; 2 Department of Health Sciences, University of York, York, UK; 3 Section of alcohol research, Institute of Psychiatry, Kings College, London, UK; 4 Institute of Health and Society, Newcastle University, Newcastle, UK; 5 Fisher Medical Centre, Skipton, UK; 6 Humber Mental Health and Teaching NHS Trust, Willerby, UK; 7 Leeds Addiction Unit, Leeds, UK; 8 Norfolk and Waveney Mental Health Foundation Trust, Norwich, UK

## Abstract

**Background:**

There is a wealth of evidence regarding the detrimental impact of excessive alcohol consumption. In older populations excessive alcohol consumption is associated with increased risk of coronary heart disease, hypertension, stroke and a range of cancers. Alcohol consumption is also associated with an increased risk of falls, early onset of dementia and other cognitive deficits. Physiological changes that occur as part of the ageing process mean that older people experience alcohol related problems at lower consumption levels. There is a strong evidence base for the effectiveness of brief psychosocial interventions in reducing alcohol consumption in populations identified opportunistically in primary care settings. Stepped care interventions involve the delivery of more intensive interventions only to those in the population who fail to respond to less intensive interventions and provide a potentially resource efficient means of meeting the needs of this population.

**Methods/design:**

The study design is a pragmatic prospective multi-centre two arm randomised controlled trial. The primary hypothesis is that stepped care interventions for older hazardous alcohol users reduce alcohol consumption compared with a minimal intervention at 12 months post randomisation. Potential participants are identified using the AUDIT questionnaire. Eligible and consenting participants are randomised with equal probability to either a minimal intervention or a three step treatment approach. The step treatment approach incorporates as step 1 behavioural change counselling, step 2 three sessions of motivational enhancement therapy and step 3 referral to specialist services. The primary outcome is measured using average standard drinks per day and secondary outcome measures include the Drinking Problems Index, health related quality of life and health utility. The study incorporates a comprehensive economic analysis to assess the relative cost-effectiveness of the interventions.

**Discussion:**

The paper presents a protocol for the first pragmatic randomised controlled trial evaluating the effectiveness and cost-effectiveness of stepped care interventions for older hazardous alcohol users in primary care.

**Trial registration:**

ISRCTN52557360

## Background

There exists a wealth of evidence regarding the detrimental impact of hazardous alcohol consumption, consuming more than the weekly recommended number of standard alcohol units in any week (21 for males, 14 for females) or half of the recommended number of standard alcohol units in any one day (10 for males, 7 for females), on the physical and mental health of the population. It is estimated that hazardous alcohol consumption accounts for 150000 hospital admissions and between 15000 and 22000 deaths per annum in the United Kingdom [1]. In the older population, those aged 55 years or more, hazardous alcohol consumption is associated with a wide range of physical, psychological and social problems. There is evidence of an association between increased alcohol consumption and increased risk of coronary heart disease, hypertension, haemorrhagic and ischemic stroke, increased rates of alcohol-related liver disease and increased risk of a range of cancers [2]. Alcohol consumption is identified as one of the three main risk factors for falls [3], a major cause of morbidity and mortality in this population. The Royal College of Physicians estimates that 60% of older people admitted to hospital because of repeated falls, confusion, chest infections and heart failure have undiagnosed alcohol problems [4]. Increased alcohol consumption in older age can also contribute to the onset of dementia and other age related cognitive deficits, Parkinson's disease and a range of psychological problems including depression and anxiety [5]. Alcohol use is implicated in one third of all suicides in the older population [6]. It is estimated that 80% of those aged 65 and over regularly take prescribed medication and polypharmacy is common with a third taking at least four prescribed medications per day [7]. Alcohol is a major contraindication for many of the drugs prescribed for older people and alcohol and medication interactions are a common phenomenon. Increased alcohol consumption in older age is also associated with a range of social problems including self-neglect, poor nutrition, social isolation and hypothermia [8].

The prevalence of hazardous alcohol consumption, this is inclusive of harmful consumption, in those aged 55 years and over is generally lower than the general population. The most recent estimate derived from the Alcohol Needs Assessment research Project [9] indicates a prevalence of between 15% and 25% and concurs with other estimates derived from the General Household Survey. There is also evidence that the prevalence rate in primary care attendees is higher than the general population [10]. There is evidence that these prevalence rates are under-estimates of the true prevalence rate. Older people are less likely to seek treatment for alcohol use disorders [11] and alcohol related presentations are often atypical or masked by comorbid physical or psychiatric illness that makes alcohol related diagnosis more difficult [12]. In 2000 16% of the UK population was over the age of 65 and this is expected to increase to 21% by 2026 [7]. As the average age of the population increases the absolute number of older people consuming alcohol at hazardous levels will increase even if the prevalence rate remains stable. Recent research using data derived from the General Practice Research Database indicates that only 5% of people aged 55 years or older with an alcohol use disorder are identified in primary care settings [13]. Opportunistic screening is a proactive screening technique that has been used with some success in a variety of healthcare areas including type II diabetes and Chlamydia [14] and is particularly useful in identifying conditions in populations who would not usually seek treatment.

A number of paper based screening methods have been developed to identify hazardous alcohol consumption; these include instruments such as the Michigan Alcohol Screening Test [15], Paddington Alcohol Test [16], Fast Alcohol Screening Test [17] and the Alcohol Use Disorders Identification Test [18]. All have acceptable levels of sensitivity and specificity. The Alcohol Use Disorders Identification Test (AUDIT) was specifically developed for use in a primary care population and has 92% sensitivity and 92% specificity for identifying hazardous alcohol use in a UK primary care setting [10]; more specifically in older populations AUDIT has been demonstrated to have higher sensitivity, 75%, and higher specificity, 97.2% than other screening tests when used in older populations [19]. AUDIT is a short 10-item questionnaire that addresses frequency of alcohol consumption, alcohol related problems and alcohol dependence symptoms. Because of the evidence of under detection and misdiagnosis of hazardous alcohol use in older populations [11,12] the proactive application of a short universal screening method is likely to be more appropriate. There is evidence that patients are more compliant with screening protocols for alcohol use in healthcare settings and that the environment provides an opportunity for a 'teachable moment' increasing the patient's likelihood to engage in an intervention [20].

There is a substantial evidence base for the efficacy of brief motivational interventions, aimed at reducing alcohol consumption in primary care. Studies have demonstrated the effectiveness of brief interventions in reducing alcohol consumption in primary care populations in the United Kingdom [21]. Further, there are six systematic reviews focusing specifically upon the effectiveness of brief interventions in primary care populations [22-27] all conclude that brief interventions in primary care populations are effective in reducing alcohol consumption. But many of the studies included in these reviews exclude older patients. There are no systematic reviews or subgroup analyses specifically focussing on older patient groups. There is some evidence from primary research of the efficacy of brief interventions specifically for older hazardous alcohol consumers. In a trial of brief interventions for older alcohol users in primary care in the United States, Fleming et al [28] reported a 34% reduction in alcohol consumption and 64% reduction in those drinking at hazardous levels at 12 months, significantly better than those who received no intervention. Blow and Barry [29] also report significantly greater reduction in alcohol use in older populations treated with brief interventions in primary care than controls. There is also evidence from subgroup analyses of existing studies that older patients are at least as likely to benefit from brief interventions as younger patients [30] and older adults are more likely to adhere and comply with brief intervention treatment regimes [31]. While a number of brief intervention studies have addressed the issue of cost-effectiveness, few have addressed the issue from a pragmatic NHS perspective. The evidence of brief interventions has been criticised for failing to address a wider range of alcohol use disorders including harmful alcohol consumption [32] and for failing to address more entrenched drinking behaviours.

Screening for alcohol use disorders identifies a range of needs that are likely to require a range of types and intensities of intervention. One of the primary reasons why many general practitioners are reluctant to implement screening into routine care is because they lack the skills of how to deal with the more severe cases identified. Motivational Enhancement Therapy is a relatively short, usually three 40 minute sessions delivered by a trained therapist, but more intensive intervention than a brief motivational intervention. Primary research has shown it to be as effective as other more intensive interventions such as cognitive behavioural therapy, twelve steps facilitation therapy and social behavioural network therapy [33,34].

Older alcohol consumers are often typified as either 'early onset' drinkers, whose consumption pattern is a continuation of lifetime hazardous consumption or 'late onset' drinkers whose alcohol consumption is a reaction to life events occurring in later life. 'Late onset' drinkers' are more likely to benefit from brief interventions than 'early onset' drinkers who often require a more intensive intervention approach [35]. Physiological changes that occur as part of the ageing process mean that older people are more vulnerable to alcohol and experience alcohol related problems at lower consumption levels than younger people. Stepped care interventions offer a potentially resource efficient means of meeting the needs of this population. Stepped care interventions provide a means of delivering more intensive interventions only to those who fail to respond to less intensive interventions and are more in keeping with rational clinical decision making than the blanket use of any one intervention strategy.

### Aims of the study

1. To evaluate the effectiveness of stepped care interventions for older hazardous alcohol users in primary care.

2. To evaluate the cost-effectiveness of stepped care interventions for older hazardous alcohol users in primary care.

3. To screen 4170 primary care attendees aged 55 years or more for hazardous alcohol use using the AUDIT questionnaire.

4. To evaluate the acceptability and validity of opportunistically screening for hazardous alcohol use in older primary care attendees.

5. To estimate the prevalence of alcohol use disorders in an older primary care population.

6. To train 15 practice nurses in the delivery of behavioural change counselling.

7. To conduct a pragmatic randomised controlled trial comparing stepped care interventions with a minimal intervention for older hazardous alcohol users in primary care.

8. To randomise 500 hazardous alcohol users, with equal probability, to either a minimal intervention or stepped care.

9. To conduct 6 and 12 month follow ups on at least 70% of those randomised to assess alcohol consumption, alcohol related problems, quality of life and service utilisation.

10. To study the process of therapy as delivered by both practice nurses and trained therapists.

## Methods/design

**Figure 1:**
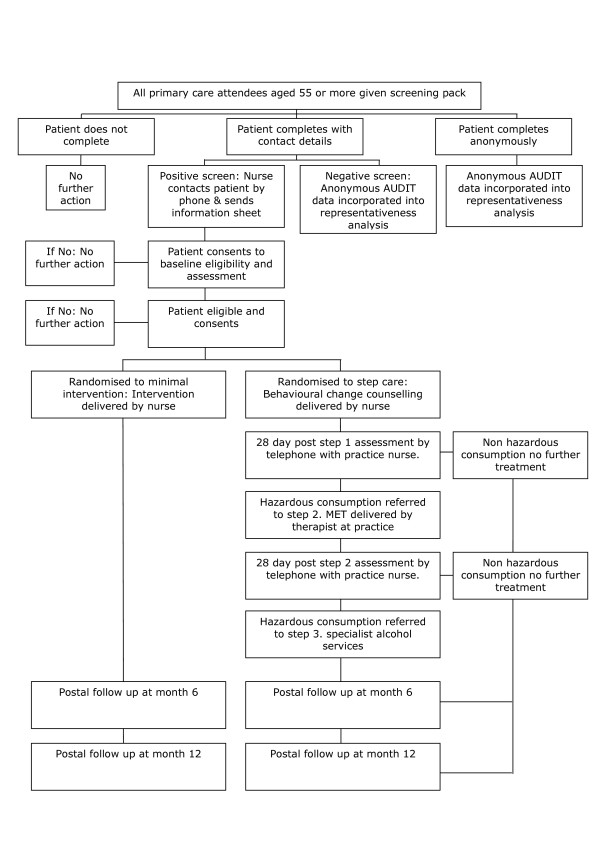
Trial Flow Chart.

The study is a pragmatic parallel group randomised controlled trial. The study has been granted ethical approval by the North West Multi-Centre Research Ethics Committee ref: 07/MRE08/24. The study complies with the Helsinki Declaration. A full flow diagram for the study is shown in figure 1.

### Hypothesis

#### Primary hypothesis

Stepped care interventions for older hazardous alcohol users reduce alcohol consumption compared with a minimal intervention 12 months after the intervention.

#### Secondary hypotheses

Stepped care is more cost-effective than minimal intervention. Stepped care will reduce alcohol related problems in the 12 months after intervention in comparison to minimal intervention. Stepped care will increase health related quality of life in the 12 months after intervention compared with minimal intervention.

### Trial inclusion/exclusion criteria

Inclusion and exclusion criteria have been chosen to maintain a balance between ensuring the sample is representative of the primary care population whilst ensuring that the trial population are able to engage both with the interventions and follow up.

#### Inclusion criteria

1. Age 55 years or over at time of screening. 2. Diagnosis of an alcohol use disorder using AUDIT criteria. 3. Residing in a stable place of residence. 4. Living within commutable distance of the primary care practice. 5. Providing informed consent for randomisation, treatment and follow up.

#### Exclusion criteria

1. Treatment for substance use in the past 90 days, excluding nicotine. 2. Already seeking help for an alcohol use disorder. 3. Received treatment for primary drug dependence, excluding nicotine in the past 90 days. 4. Outstanding legal issues likely to lead to imprisonment. 5. Severe mental or physical illness likely to preclude active participation in treatment or follow up.

### Randomisation and consent

#### Screening

In accordance with guidance on best practice, all attendees at primary care who are aged 55 years or more will be informed that a study is taking place. They will be provided with an information letter and a copy of the AUDIT questionnaire. The information letter will provide details of the study taking place and make clear that completion of the screening questionnaire is not compulsory. Participants will have the option to not complete the questionnaire, to complete the questionnaire anonymously or complete the questionnaire with full contact details. Completed questionnaires will be returned to the practice in sealed envelopes or directly to the study co-ordinating centre in York.

#### Invitation to attend practice nurse assessment

For all AUDIT positives who complete their contact details and wish to take part in the study, a standard baseline assessment will be completed with all information recorded on forms containing only an identification number. Interested patients will be telephoned by the practice nurse and an appointment made for them to attend the practice. A detailed information sheet providing information on the purpose of the study, the proposed interventions and follow up assessments will be sent to the potential participant, stating that participation is not compulsory.

#### Baseline assessments

At the assessment the practice nurse will discuss the study, providing the potential participant with an opportunity to ask any questions about participation in the study, and assess further eligibility. Eligible participants will be invited to provide written informed consent. For those who do consent, randomisation will be conducted using the secure remote randomisation service at York Trials Unit. At this point the patients contact details and identification number will be associated and held on a secure server located at the University of York. This master register will be held separate from the outcome data and accessible only to those who need to know for purposes of conducting the study. Randomisation will be conducted using block randomisation stratified by cluster with an equal probability of receiving stepped care or minimal intervention.

### Interventions

#### Screening

All primary care attendees, aged 55 years or older, will be provided with an information letter, a copy of the AUDIT questionnaire and a return envelope addressed to the study co-ordinating centre. Returned questionnaires, enclosed in a sealed envelope, will be scored by the practice nurse by summing the responses to all 10 questions on the AUDIT questionnaire, or returned directly to the co-ordinating centre for scoring. Patients who score 8 or more on the AUDIT questionnaire, who are willing to be contacted and complete a baseline assessment will be invited to a research assessment with the practice nurse. At the research assessment the research nurse will explain the study, provide an opportunity to ask any questions and ask the potential participant for informed consent. The research assessment will include a check on eligibility. If consenting, the patient will be randomised using a remote randomisation service, with equal probability to either minimal intervention or stepped care.

#### Minimal Intervention

The minimal intervention consists of a short, 5 minute, discussion with the practice nurse about the health consequences of continued hazardous alcohol consumption. The participant will also receive a brief self-help leaflet 'Safer drinking – a self help guide' outlining the consequences of excessive alcohol consumption and providing information on sources of help for drinking problems locally and nationally.

#### Stepped Care Intervention

The stepped care intervention consists of three consecutive steps in which progression between steps are dependent upon the outcome of each previous step.

Step 1 will consist of a 20 minute session of behavioural change counselling delivered by the practice nurse. This intervention, based upon an existing evidence base of brief interventions, utilises the technique of motivational interviewing [32] and aims to address the individual's motivation to change their drinking behaviour. The counselling is manual guided and practice nurses will be trained in the delivery. Four weeks after the step 1 assessment the participant will be contacted by the practice nurse and a short telephone assessment will be made about the participant's alcohol consumption in the past 4 weeks using the extended AUDIT-C. If the participant is still consuming alcohol at hazardous levels a referral will be made to step 2 of the intervention.

Step 2 involves an intervention by a trained alcohol therapist in the primary care environment. The intervention, Motivational Enhancement Therapy, is provided through three, 40 minute sessions on a weekly basis. The intervention is manual guided and addresses six basic principles of increasing motivation for change. Feedback about individual alcohol consumption, emphasis on the individual as being the agent responsible to change, advice on how to accomplish change, provision of alternative vehicles for change, maintenance of an empathetic therapeutic style and emphasis on enhancing the individuals self-efficacy. Four weeks after the last MET session the participant will be contacted by the practice nurse and a short telephone assessment will be made about the participant's alcohol consumption in the past 4 weeks using the extended AUDIT-C. If the participant is still consuming alcohol at hazardous levels a referral will be made to step 3 of the intervention.

Step 3 will consist of a referral to the local specialist alcohol services to receive specialist intervention, including as necessary detoxification, inpatient care, outpatient counselling, group therapy, relapse prevention treatment or medication. There is no limit on the intensity or duration of the step 3 intervention.

Particular emphasis is being paid to ensure that the interventions are pragmatic in nature. The interventions will be delivered by staff routinely employed in primary care, in the case of practice nurses, and specialist alcohol services in the case of motivational enhancement therapists. All of the interventions will be manual guided to specify the purpose and principles of each intervention and the structure and content of each particular treatment session.

#### Training of practice nurses to deliver behavioural change intervention

It is proposed to train 15 practice nurses in the techniques and delivery of a brief motivational behavioural change intervention. Each practice nurse will spend 2 days at the training centre at Leeds Addiction Unit. Training will be provided by expert trainers in motivational interviewing. The training will take the form of simulated consultation/seminar/simulated consultation. Each nurse will have the opportunity to engage in a simulated consultation which is recorded. As a group the nurses will discuss the simulated consultations to examine and review application of motivational interviewing techniques. Prior to embarking on the study assessment of competency will be made using a recorded session rated by an independent expert. Practice nurses will be provided with ongoing supervision throughout the study provided by an expert trainer from Leeds Addiction Unit. A further training day is provided covering protocol issues and use of the study database.

#### Training of therapists to deliver Motivational Enhancement therapy

It is proposed to train alcohol therapists from local alcohol agencies. Therapists will have at least two years post-qualifying experience. Initial training will involve a three day intensive group training course provide by motivational enhancement trainers at Leeds Addiction Unit. Particular attention will be given to understanding the evidence base, understanding the theoretical basis of treatment, demonstration of practice and role-play opportunities. Therapists will be supervised in the delivery of a number of therapy sessions. Therapists will be expected to complete two taped sessions both reviewed in conjunction with a trained supervisor. Supervision will provide the main opportunity for practising skills and delivering the structure and content of treatment. Assessment of competence will depend upon the therapist's ability to deliver motivational enhancement therapy according to the designation of treatment prescribed in the treatment manual.

### Outcome measures

#### Screening

Screening for alcohol use disorders will be conducted using the Alcohol Use Disorders Identification Test (AUDIT) [18]. The instrument addresses alcohol consumption frequency and quantity, alcohol related problems and elements of alcohol dependence. The 10-item patient completed questionnaire takes approximately 3 minutes to complete and 2 minutes to score. A score of 8 or more indicates hazardous alcohol use. AUDIT exhibits high levels of sensitivity (92%) and specificity (92%) in UK primary care populations [10] and high levels of sensitivity (75%) and specificity (93%) in older populations [19].

#### Eligibility assessment

To establish eligibility a potential participant should score positive for the AUDIT questionnaire and be classified as a hazardous alcohol user using extended AUDIT-C criteria. Hazardous alcohol consumption is established if the participant has consumed more than 21 standard units for males, or 14 for females, in any one week or 10 standard units for males or 7 standard units for females in any 1 day in the previous 90 days. The extended AUDIT-C is used to derive the primary outcome measure for the study.

#### Primary outcome measure

The primary outcome measure for the study is average drinks per day, where a standard drink equates to 8 mg of ethanol. This is ascertained using the extended AUDIT-C. Three other variables can be derived from the data; percent days abstinent, drinks per drinking day and total alcohol consumed. The extended AUDIT-C is self-completed and takes approximately 2 minutes to complete. The outcome is measured at baseline, 6 months post randomisation and 12 months post-randomisation.

#### Secondary outcome measures

1. Alcohol related problems measured at baseline, 6 months and 12 months post randomisation. Alcohol related problems are assessed using the 17-item participant completed Drinking Problems Index (DPI). The DPI has been specifically designed and validated for use in older populations [36]. 2. Quality of life is measured at baseline, 6 months and 12 months post randomisation. Quality of life is measured using the SF-12 [37]. SF-12 is a 12-item self completed questionnaire that established validity and reliability for measuring physical health and mental health components of quality of life. 3. Health utility will be measured at baseline, 6 months and 12 months using the EQ-5D [38]. EQ-5D is a 5-item participant completed questionnaire with established reliability and validity in this population.

#### Economic outcome measures

Opportunistic screening costs will be estimated from the actual costs of screening using the actual costs of screening associated with the study. Costs of delivering the minimal intervention and the first two tiers of stepped care will be based upon actual patient contact time from time sheets maintained by practice nurses and therapists. The units of services used will be based upon local costs of services and include allowances for managerial and premises overheads and the costs associated with training and supervision using methods utilised in similar intervention studies [39]. The costs of any specialist referral will be ascertained using information on the actual costs associated with specialist service provision based upon Department of Health costs of specialist interventions [40]. Participant use of health services, other alcohol services outside the study, public services and criminal justice services will be assessed using a service use questionnaire at baseline, 6 months and 12 months post randomisation. The service use questionnaire has been developed over a number of alcohol intervention studies [39,41] will be adapted to capture costs specifically associated with this population.

#### Quality assurance of treatment delivery

Participants will be asked to provide consent to have all treatment sessions tape recorded. A 20% sample of each type of treatment session, minimal intervention, behavioural change intervention, motivational enhancement therapy will be randomly selected stratified by treatment type. Tapes will be rated by an independent rater and assessed for quality of delivery and compliance with treatment protocols.

#### Sample size calculation

There are no previous studies of stepped care interventions, a brief opportunistic intervention followed by successively more intensive interventions for those who fail to respond to treatment, for older alcohol using adults. The closest UK pragmatic randomised controlled trials include Wallace et al [21] and STEPWICE [41], both of these reported effect size differences between stepped care and minimal intervention of 0.36 and 0.27 respectively. Similar effect size differences are reported in studies from the United States [29,42,43]. There is evidence that older populations respond as well, or even better, to brief psychosocial interventions for alcohol use than general populations [31,44]. Assuming a conservative effect size difference between stepped care and minimal intervention of the order of 0.3 would require a sample size of 175 participants in each of the two randomised groups, using power at 80% and a 5% significance level.

Our previous experience in conducting randomised controlled trials in the fields of substance use, alcohol using populations [34,41] and elderly populations indicate that with assiduous follow up regimes loss to follow up at 12 months is of the order of 20%. There also exists evidence that older populations are more compliant with treatment regimes and follow up protocols than younger populations [45]. Taking these factors into account we have erred on the side of caution and allowed a loss to follow up of 30%, requiring 500 participants to be randomised, 250 in each group. Previous alcohol use screening and intervention studies conducted in UK healthcare settings [46] suggest that 80% of those screened positive tend to be eligible and 75% of those eligible tend to consent to randomisation. This means the study requires 834 screen positives of whom we predict 500 will be eligible and consent to randomisation.

The prevalence of hazardous alcohol consumption, inclusive of harmful consumption, in those aged 55 years or older is estimated at 15% in the general population [9] and greater, at 25%, in those attending primary care [10]. If we conservatively estimate the prevalence at 20% we would need to screen 4170 primary care attendees in an 18 month period. Assuming 15 practices, in three geographic regions consent to take part in the study, each practice would be expected to screen 278 primary care attendees over 18 months, a total of 18 per practice per month.

### Statistical analysis

#### Opportunistic screening

We will use a comprehensive cohort approach to the analysis of the acceptability and validity of opportunistic screening. Participants will have a choice of not completing the questionnaire, completing the questionnaire with basic age/sex demographics or completing the questionnaire with full contact details.

#### Effectiveness analysis

The primary analysis will be intention to treat comparing minimal intervention with stepped care on the primary outcome measure, average drinks per day, at 12 months post-randomisation. Participants will be analysed as part of the group allocated irrespective of treatment received. The primary outcome will be analysed using analysis of covariance controlling for baseline values. Multi-level modelling analysis will be undertaken to account for any variation due to centre, cluster and therapist. Primary analysis will be conducted after all 12 month follow ups have been completed. Analysis of secondary outcomes will be conducted using analysis of covariance and adjusted using multi-level modelling. Regression analysis will be undertaken to explore any baseline predictors of outcome, any baseline predictors of referral to step 2 for the stepped care group and any potential baseline × treatment interaction effects.

#### Economic analysis

The incremental cost-effectiveness of stepped care compared to the minimal intervention will be assessed both from a health and personal social services perspective following NICE guidance [47] and a wider public sector resource perspective [48]. While the opportunistic screening costs will be common to both intervention arms, its cost will be estimated from the trial data as this would form part of a wider implementation cost of the stepped care programme. The costs of the minimal intervention and the first two tiers of the stepped care programme will be based on information gathered on patient contact with the primary care and specialist services during the trial. The units of service used will be based on the local costs of specialist services and include an allowance for the training and supervision costs, using methods developed for the UKATT trial [39]. Utilisation of more specialist services will be collected, including the type of intervention, and costs will be applied from previous research trials and a current Department of Health funded research project based on a range of specialist providers and intervention types [40]. The use of alcohol services outside the trial protocol, along with all other public sector services, including health, social welfare and contact with criminal justice agencies will be assessed from questionnaires administered at baseline, 6 and 12 months. This service use questionnaire was developed over a number of alcohol and illicit drug trials and has been adapted for the specific needs of this project, for example, by including additional questions on falls. Units of resource use recorded will be combined with national sources of unit costs [49,39] which will provide generalisable results. The EQ-5D will be used with population values and the QALY change calculated using the area under the curve method [50]. Bootstrapping methods will be used to test to explore the sensitivity of the calculated incremental cost-effectiveness ratios and cost-acceptability curves presented [51].

#### Frequency of analysis

Analysis will be conducted after the final 12 month follow up has been completed.

#### Ethics and confidentiality

The study has been granted ethical approval by multi-centres research ethics committee and by the local research ethics committee for the localities where the research will take place. There are no anticipated risks in relation to either treatment. There is no documented evidence of adverse events arising due to either the minimal intervention or the stepped care intervention.

All trial data will be identified using a unique trial identification number. No personally identifiable information will be held beyond the final 12 month follow up. Analytical datasets will not contain any patient identifiable information. Anonymised data will be retained for a period of 42 months.

## Competing interests

The authors declare that they have no competing interests.

## Authors' contributions

SC is the chief investigator for the study. All authors contributed to the design and continuing management of the study. All authors reviewed and contributed to successive drafts of the paper. SC prepared the final draft for publication.

## Pre-publication history

The pre-publication history for this paper can be accessed here:


